# Supplementary material for: In Drosophila Hemolymph, Serine Proteases Are the Major Gelatinases and Caseinases
Source: Insects. 2024 Mar 28;15(4):234. doi: 10.3390/insects15040234 (PMC11050137; doi:10.3390/insects15040234)
Supplement: Supplementary file 1 [file insects-15-00234-s001.zip › supplementary /Gatti et al Supplementary materials V2.pdf]

Gatti et al., In Drosophila hemolymph, serine proteases are the major gelatinases and caseinases. Supplementary materials

Figure S1: Control of potential contamination of hemolymph gelatinases.
A, gelatin SDS-PAGE separation of *D. melanogaster* Nasrallah L2 larvae hemolymph shown as reference (from Fig. 1). The white bands after Coomassie blue staining indicate the gelatinase activity.
B, gelatinases of total larval extracts from 20 L1, 10 L2, 5 L3 larvae *D. melanogaster* Nasrallah and Canton S strains.
C, protease inhibitors effect on total L2 larval extract of *D. melanogaster* Nasrallah. L2 total larval extract from 20 larvae was loaded on the top of the gel and after migration the gel was sliced and incubated in PBS (control) or in PBS and the indicated inhibitor (inhibitor cocktail contained AEBSF, Bestatin, E-64, Pepstatin A, Phosphoramidon, Leupeptin, Aprotinin).
D, gelatinase activity in the ringer solution after dipping 20 intact L2 larvae *D. melanogaster* Nasrallah in Insect Ringer for 10 minutes.
E, gelatinase activity in the ringer solution after centrifugation of intact *D. melanogaster* Nasrallah L2 larvae (20 larvae).
F, comparison of the gelatinase activity between the hemolymph and the gut from *D. melanogaster* Nasrallah L2 larvae (hemolymph from 15 larvae, gut extract equivalent to 0.5 larvae in order to visualize the bands).
12.5% gelatinase SDS PAGE. MW in kDa.

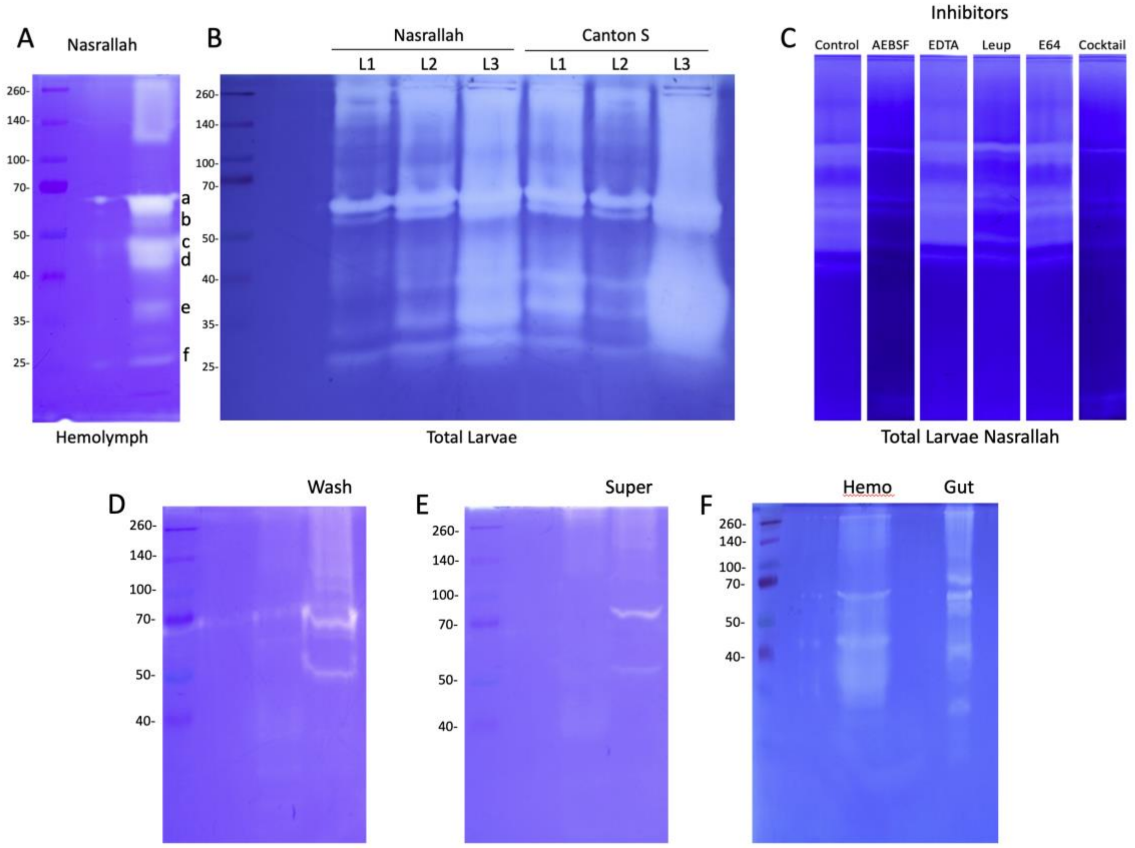

Figure S2: Hemolymph gelatinases from *D. melanogaster* strains and Drosophila species.

A) Hemolymph from 10 L2 larvae from *D. melanogaster* strains Nasrallah, Canton S, YS, YR, Sefra and Brazza was separated on 12.5% gelatinase SDS PAGE. MW in kDa.

B) Hemolymph gelatinases from *D. melanogaster* Nasrallah (Nas) and Canton S (CanS) strains were compared with those of *D. suzukii* (Suz), *D. yakuba* (Yak), *D. immigrans* (Imm) and *D. simulans* (Sim). Hemolymph from 10 L2 larvae separated on 12.5% SDS-PAGE. The high level of LSPs present in *D. suzukii* hemolymph suggests that the larvae were almost at the L3 stage and impaired the visualization of the 40 kDa gelatinase band.

A)

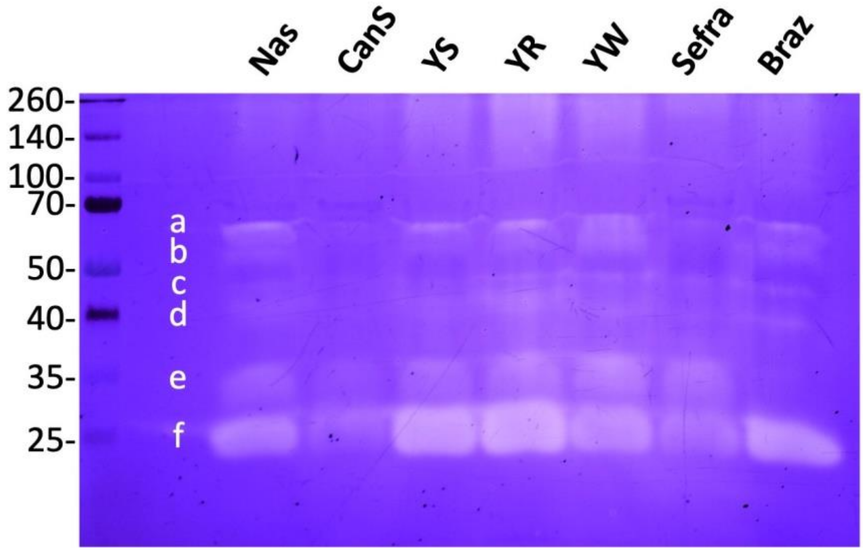

B)

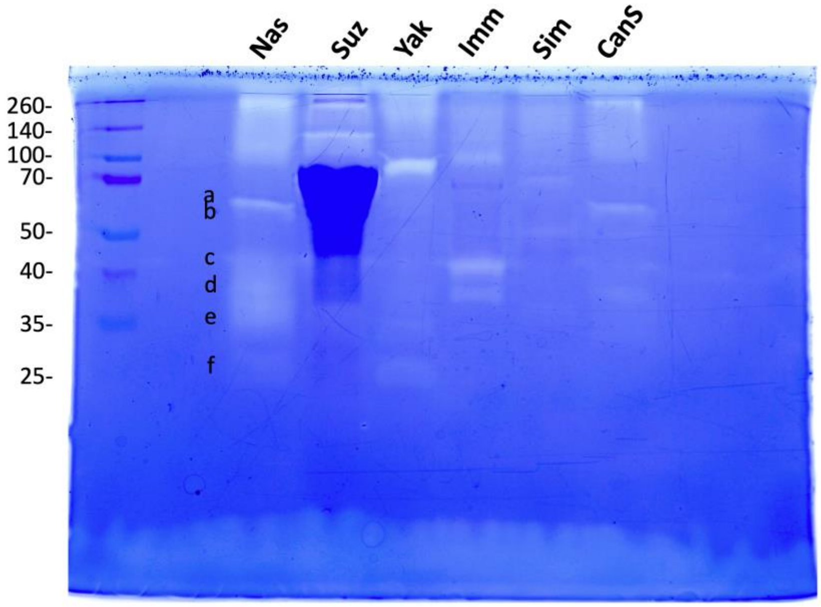

Figure S3: Effect of beta-mercaptoethanol on hemolymph proteins and gelatinases.
A, hemolymph from 20 L2 larvae from *D. melanogaster* Nasrallah was mixed with sample buffer with an increasing concentration of the reducing agent beta-mercaptoethanol and separated on SDS PAGE that was silver stained (A) or a gelatin gel was done with equivalent samples (B). C, effect of the presence of 5% beta-mercaptoethanol on the migration of hemolymph proteins from L2 larvae (10 L2) from different Drosophila species (*D. melanogaster* Canton S (CanS) et Nasrallah (Nas); *D. suzukii* (Suz); *D. yakuba* (Yak); *D. immigrans* (Imm); *D. simulans* (Sim). 12.5% SDS PAGE. MW in kDa.

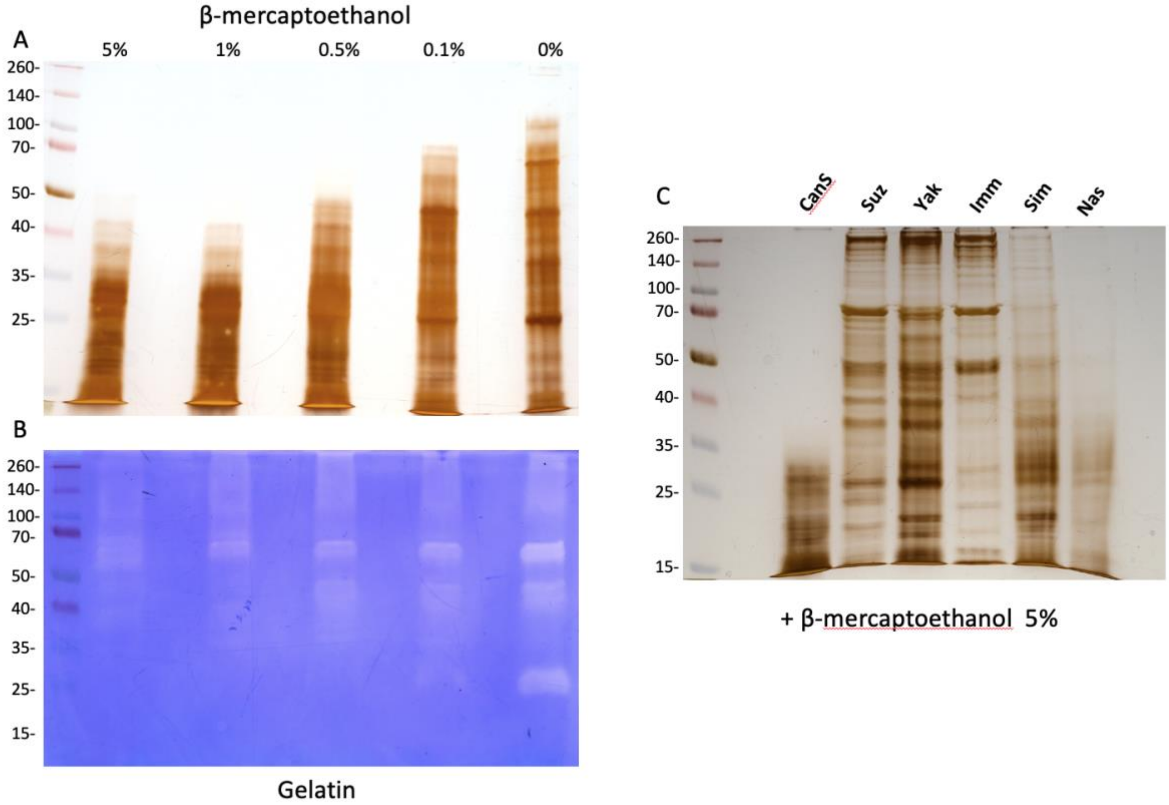

Figure S4: Effect of AEBSF on hemolymph gelatinase and caseinase activity from *D. melanogaster* Nasrallah strain.

Hemolymph from 20 L2 larvae was separated either on a gelatin or a casein gel and for each gel one lane was incubated with PBS and one with PBS and AEBSF.

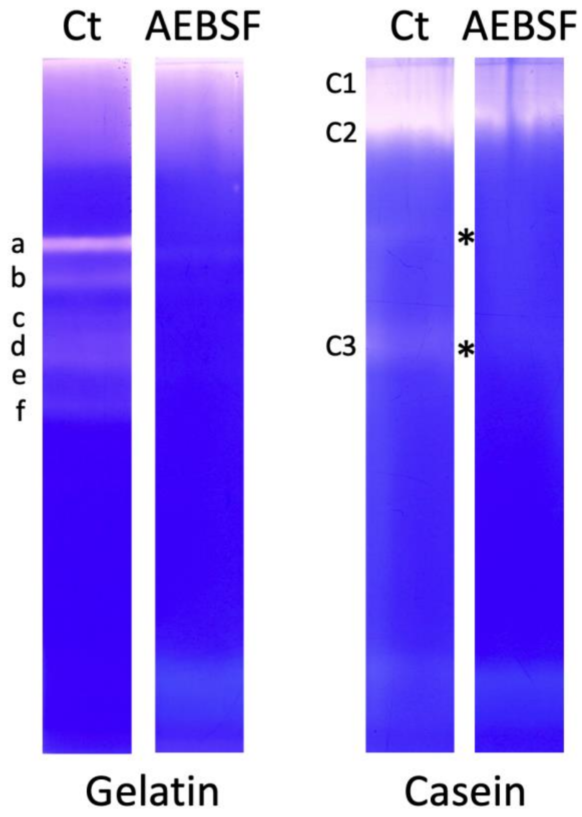

Figure S5: Comparison of the main hemolymph gelatinases from *D. melanogaster* Nasrallah and Canton S strains.

Hemolymph from 10 L2 larvae from each *D. melanogaster* strain was separated on a gelatin or on a normal gel. The framed silver-stained bands corresponding to the gelatinase bands (stars) were cut and used for mass spectrometry (see Table S2).

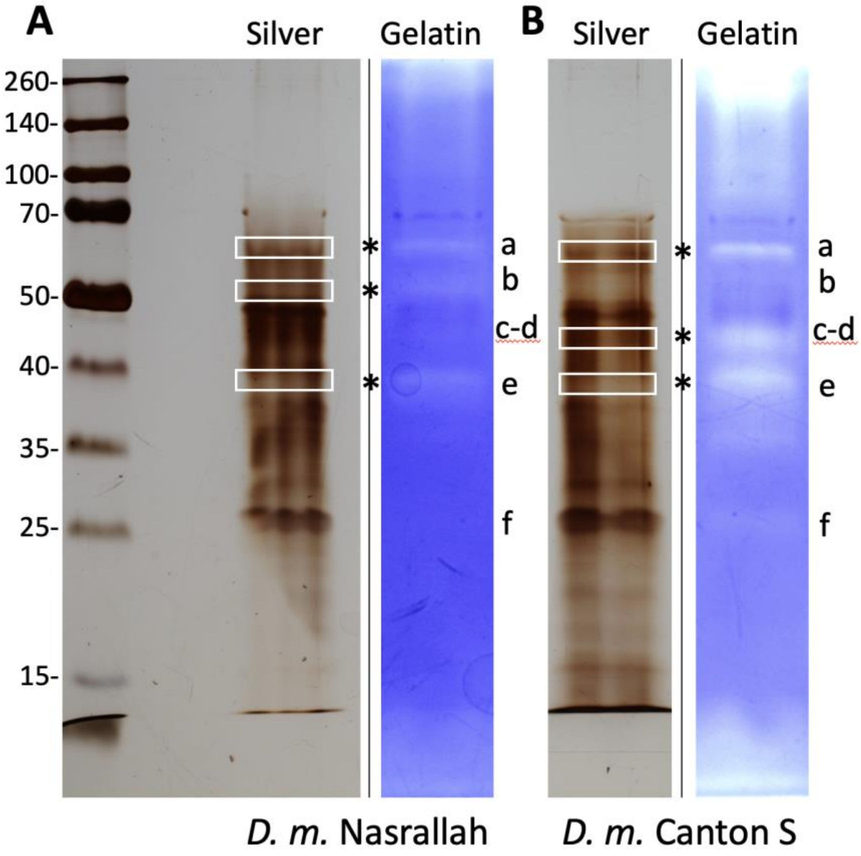

Figure S6: Separation of *D. melanogaster* hemolymph by two dimensional SDS-PAGE. Hemolymph from 100 Nasrallah L2 larvae was separated by two-dimensional electrophoresis in non-denaturation conditions either on a normal gel that was further silver stained and on a gelatin gel that was further incubated to reveal the gelatinases. The framed area on the silver-stained gel corresponding to the main visible gelatinase spot at about 50 kDa was cut and submitted to mass spectrometry (see main text). 12.5 % SDS PAGE. Ac, acid side of the gel; Bas, basic side of the gel.

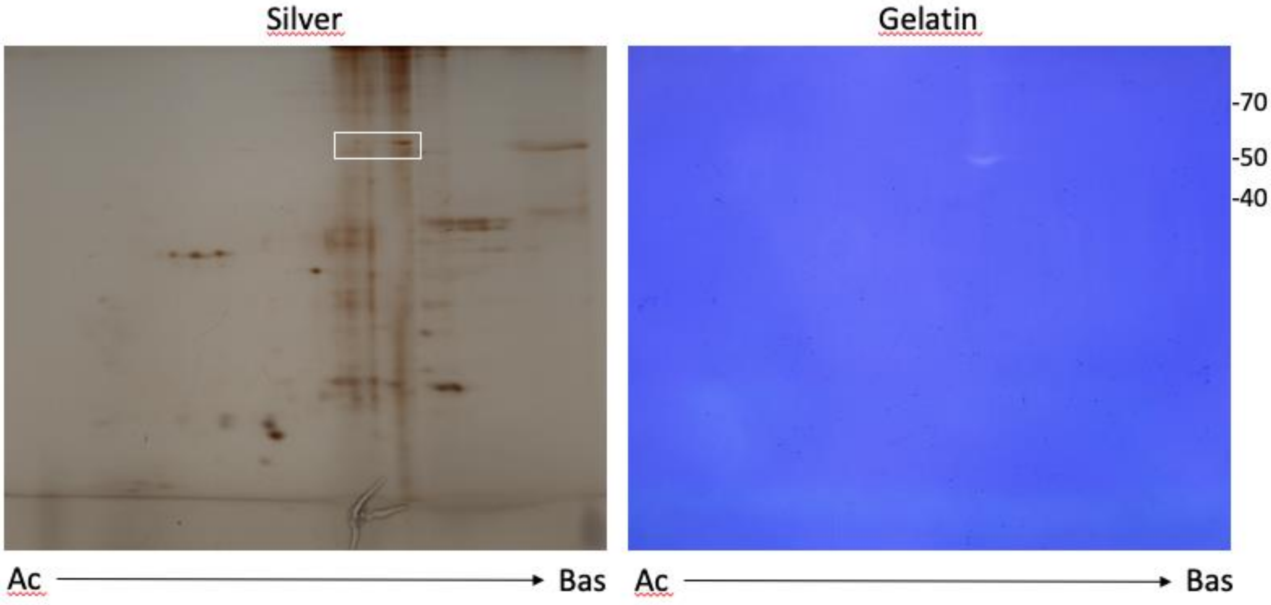

Figure S7: Purification of *D. melanogaster* hemolymph gelatinases.

Hemolymph from *D. melanogaster* Nasrallah L2 larvae was separated either on DEAE-sephadex, gelatin-sephadex or benzamidine-sepharose beads as described in Material and Methods. The collected fractions were separated by normal SDS-PAGE (12.5%) and silver stained (upper panels) or by gelatin containing SDS-PAGE (lower panels).

The framed bands from the DEAE silver stained corresponding to the ~30 kDa gelatinase and the numbered bands from the Benzamidine-Sepharose PABA and SB elution lanes were submitted to mass spectrometry (see Table S1). (PABA, para-aminobezamidine; SB, indicates beads eluted with non-reducing sample buffer).

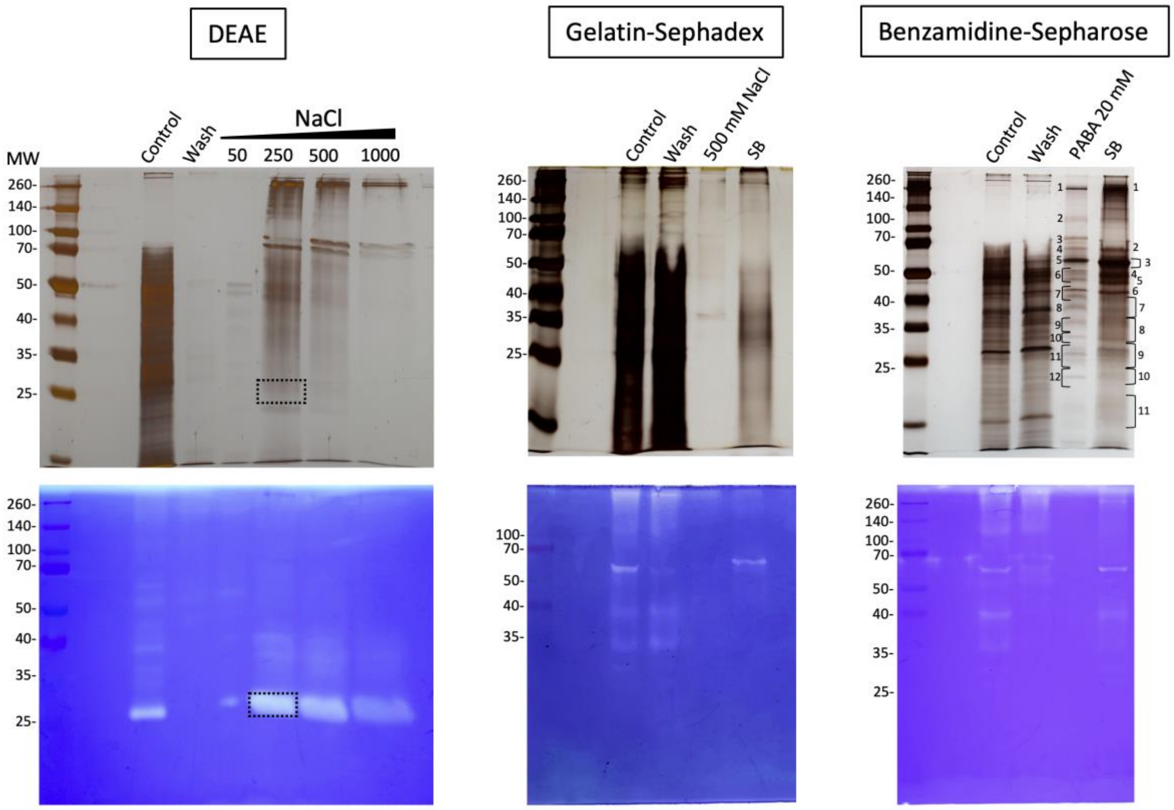

Figure S8: Comparison of *D. melanogaster* Nasrallah and Iso1 strains hemolymph gelatinases.
Left panel: PCR showing the absence of *tequila* expression in the Iso1 strain (cDNA from L2 larvae). *RP49* (*Ribosomal protein L32*) used as a cDNA quantity control. Amplicons were visualized and photographed on a 2% agarose gel after ethidium bromide staining.
Right panel: 2 separated gelatinase gels with the hemolymph from 25 *D. melanogaster* Nasrallah (NasR) and 25 *D. melanogaster* Iso1 L2 larvae. SDS-PAGE (12.5%). MW in kDa.

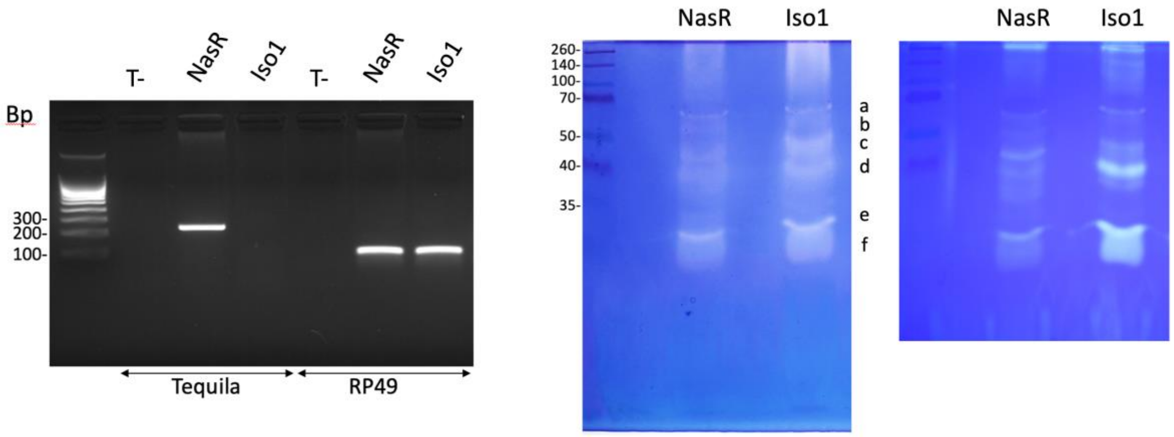

Figure S9: *D. melanogaster* YR hemolymph gelatinases after parasitism or mixture with parasitoid venom.

A, gelatinase gel with the hemolymph from 20 *D. melanogaster* Nasrallah (NasR) and YR 24h after parasitism with *L. boulardi* ISm or ISy (see materials and methods). Control, hemolymph from 20 *D. melanogaster* YR larvae from the same age. SDS-PAGE 10%. MW in kDa.

B, gelatinase gel with the hemolymph from 10 *D. melanogaster* Nasrallah (NasR) L2 larvae either incubated for 1h alone or with venom extract from 5 females L. *boulardi* ISm venom reservoir (result was similar with ISy venom). Venom alone as no gelatinase activity (not shown). SDS-PAGE 12.5%. MW in kDa.

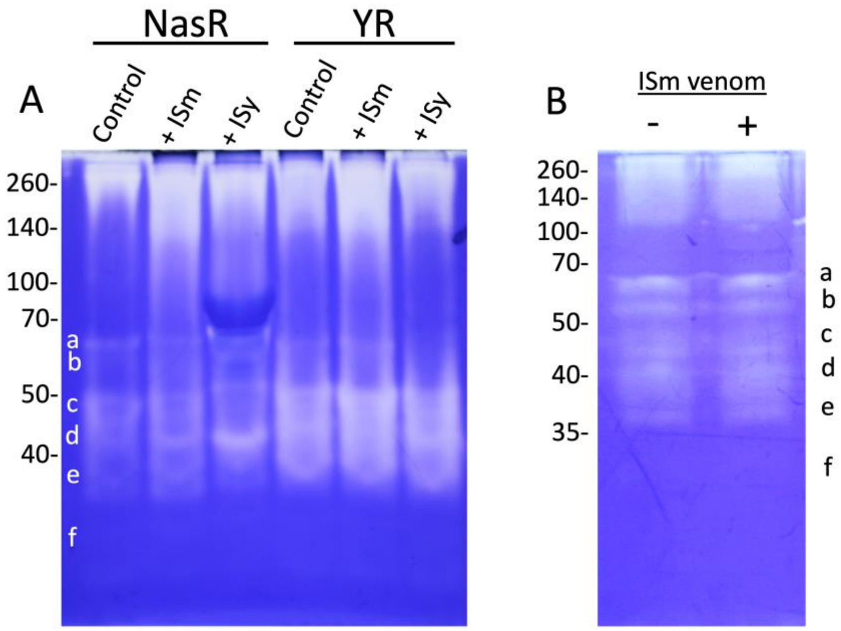

Table S2. Statistical data for the bands a, b, d, f for the gelatinase inhibition and a and d for LPS injections. n=3. (nd, not measured**)**

Tukey's multiple comparisons test	Adjusted P Value			
**Inhibition**	band a	band b	band d	band e
Control vs. E64	0.7689	0.4866	0.4264	0.1272
Control vs. AEBSF	**0.0012**	**0.0129**	**0.0027**	**0.0042**
Control vs. Leup	**0.0044**	**0.0069**	**0.0185**	**0.0036**
Control vs. EDTA	0.8761	>0.9999	>0.9999	0.9783
**LPS injection**				
Control vs. Ser	0.984	nd	nd	0.9918
Control vs. Sal	>0.9999	nd	nd	0.9962
Control vs. Ec0111	>0.9999	nd	nd	>0.9999
Control vs. Ec055	0.9995	nd	nd	0.9743

Table S3. Proteases identified by MS-MS in the gel bands from hemolymph of *D. melanogaster* Nasrallah and Canton S strains.

**Nasrallah**	**Canton S**
MP1 (149)	SP7 (222)
Tequila (109)	Jon25Bi (212)
Jon65Aiii (51)	MP1 (120)
SP7 (66)	Tequila (85)
Hayan (38)	SP34(CG9372) (67)
	SPE (62)
	Grass (59)
	Hayan (46)
	SP91(CG16749) (41)
	Jon65Ai (41)

The indicated bands from the gel in Fig. S5 were submitted to MS-MS. In parenthesis the best mascot score associated to the protein.
